# Network inhomogeneity supports burst initiation in vitro

**DOI:** 10.1186/1471-2202-12-S1-P84

**Published:** 2011-07-18

**Authors:** Samora Okujeni, Nila Moenig, Steffen Kandler, Oliver Weihberger, Ulrich Egert

**Affiliations:** 1 Bernstein Center Freiburg, Univ. Freiburg, Freiburg, Germany; 2 Fac. Biol., Univ. Freiburg, Germany; 3 Fac. Engineer. – IMTEK, Univ. Freiburg, Germany

## 

The emergence of spontaneous bursting events in developing neuronal networks likely depends on the evolving network connectivity. Theoretical models have shown that hierarchical network structures embedding clusters of strongly inter-connected neurons are optimal for initiating and sustaining spontaneous activity [1]. It is conceivable that activity-dependent wiring could innately support the formation of similar network structures.

**Figure 1:**
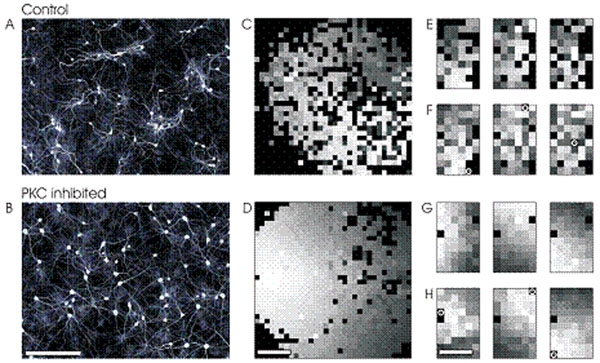
MAP2 staining of dendrites and somata: control networks (A) display characteristic features of an activity-dependent wiring process including fasciculation and ramification of dendrites within clusters. Developmental inhibition of PKC (B) generates more homogeneous networks in which neurites grow out ignoring neuronal neighbors. Propagation of activity in spontaneous and stimulation-induced bursts was analyzed in micro-electrode recordings (first spike rank order from light to dark gray; black: no activity; white circles indicate stimulation sites): bursts in control networks (C) display irregularities in the spatial propagation pattern indicating complex underlying structure. Homogeneous networks developing under impaired PKC activity (D) show highly isotropic propagation patterns. Spontaneous (E) and elicited (F) propagation patterns in controls are hardly comparable. Stimulation of homogeneous networks elicits highly isotropic propagation patterns (H) similar to those in spontaneous bursts (G). Scale bars: 2mm.

To test this we chronically manipulated activity-dependent structural plasticity by inhibition of protein kinase C (PKC) in developing networks of cortical neurons in vitro. Previous studies showed that PKC inhibition in developing cerebellum promotes dendritic outgrowth and arborization of Purkinje cells and impairs pruning of climbing fibers. We found that developmental inhibition of PKC in cortical cell cultures increased dendritic outgrowth, impaired neurite fasciculation and clustering and abolished network pruning. This resulted in more homogeneous and potentially better connected networks (fig. 1A-B). As a result, propagation of activity within bursts was faster and occurred in strongly isotropic waves (fig. 1C-D). Interestingly, bursts in these networks were triggered from fewer sites and at much lower rates suggesting that the homogeneous networks forming under blockade of activity-dependent wiring processes embed fewer burst initiation zones.
